# Association between vitamin intake and prostate cancer: a cross-sectional study

**DOI:** 10.3389/fnut.2025.1607452

**Published:** 2025-06-13

**Authors:** Sen Pan, Chuanlin Wang, Wei Sun, Xin Zhang

**Affiliations:** Department of Urology, Chongqing University Fuling Hospital, Chongqing, China

**Keywords:** vitamin intake, prostate cancer, cross-sectional, National Health and Nutrition Examination Survey, diet

## Abstract

**Background:**

As micronutrients, vitamins play a critical role in maintaining normal physiological functions. However, the impact of different types of vitamins on PCa remains controversial. This study aimed to investigate the association between vitamin intake and PCa using a cross-sectional design.

**Methods:**

We conducted a cross-sectional analysis of 14,977 adult men using data from the National Health and Nutrition Examination Survey (NHANES) collected between 2007 and 2018. Dietary intake was assessed using 24-h dietary recall interviews. Multivariate weighted logistic regression models were used to analyze the relationship between vitamin intake and PCa. Restricted cubic spline (RCS) was conducted to evaluate the non-linear relationship. We performed a trend test to examine the association between vitamin intake and PCa risk, and conducted an interaction analysis stratified by group covariates. The covariates included age, race, body mass index, educational attainment, the ratio of family income to poverty, alcohol intake, smoking status, diabetes, and hypertension.

**Results:**

The study encompassed 10 vitamins with three ways of intake: diet, supplement, and total (diet plus supplement). In the fully adjusted model, the quartile-based analysis showed that individuals in the highest quartile of dietary retinol intake had a significantly increased risk of PCa (OR = 1.76, *p* = 0.027), while higher supplement intake of vitamin B1 (OR = 0.38, *p* = 0.036) and vitamin B2 (OR = 0.35, *p* = 0.016) was associated with a lower risk. In the continuous variable analysis, supplement intake of vitamin B9 (OR = 0.65, *p* = 0.049), vitamin B12 (OR = 0.83, *p* = 0.030), and total vitamin B12 (OR = 0.82, *p* = 0.037) were inversely associated with PCa risk after full adjustment. We identified significant non-linear associations between dietary intake of vitamins A, B6, B12, and C and PCa risk using RCS analysis. There is an interaction between supplementation, total vitamin B12 intake, and age groups.

**Conclusion:**

Taken together, our study provides the latest evidence for vitamin intake and PCa prevention. Large-scale randomized controlled trials are still needed to provide additional evidence.

## 1 Introduction

Prostate cancer (PCa) ranks as the fifth highest number of cancer-related fatalities among men worldwide, with an estimated annual occurrence of around 1.3 million cases (1, 2). Despite advancements in early screening, regions such as Asia and South America continue to grapple with elevated PCa mortality rates (2). The progression of PCa is a highly intricate process, often spanning decades from the initiation of pathological changes to the manifestation of clinical symptoms (3). Several studies have identified associations between PCa development and factors such as age, race, metabolic syndrome, diabetes, and obesity (3–6). However, the latest study from the European Association of Urology on dietary factors found no conclusive evidence supporting an association between specific dietary factors and PCa development (7).

Vitamins are small-molecule organic compounds that complement the three macronutrients: carbohydrates, proteins, and lipids (8). Although vital for maintaining physiological homeostasis, many vitamins cannot be synthesized by the human body and must be obtained through diet or supplementation. However, evidence regarding the relationship between various vitamin intake and PCa risk remains insufficient, particularly for families such as the B vitamins (7, 9). For instance, the association between vitamins B9 and B12 and PCa remains controversial across different studies (10–13). Existing research on the relationship between vitamin A intake and PCa risk is scarce and has produced conflicting findings (14, 15). The effects of other vitamins are also inconclusive. Vitamin C, widely recognized for its antioxidant properties, may offer anticancer benefits, yet excessive intake may paradoxically promote oxidative stress (9). A dose–response meta-analysis by Gao et al. suggested an association between elevated circulating concentrations of 25-hydroxyvitamin D and increased PCa risk (16). Conversely, Pernar et al. (17) proposed that vitamin D may reduce PCa risk based on existing evidence. Similar controversies surround the effect of vitamin E and others on PCa risk (18–20). Although some *in vitro* and *in vivo* studies have demonstrated the antitumor activity of vitamin K, certain clinical and basic studies have also reported antitumor properties of vitamin K antagonists (21, 22).

The National Health and Nutrition Examination Survey (NHANES) provides data that accurately reflects the health and nutrition status across the nation, employing a multistage, stratified, randomized sampling approach. Hence, we undertake observational studies utilizing NHANES data to assess the association between vitamin intake and PCa risk, providing valuable recommendations for vitamin intake and PCa prevention.

## 2 Methods

### 2.1 Cross-sectional study design

**Figure 1:**
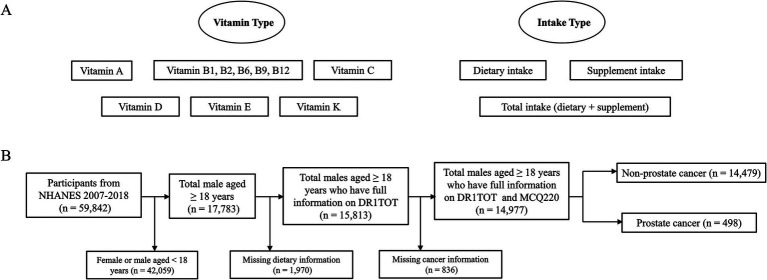
Study design and process. **(A)** The 10 types of vitamins and three types of intake in this study. **(B)** The flow chart of participants selection in the cross-sectional study. NHANES, National Health and Nutrition Examination Survey. DR1TOT, the total nutrient intake on the first day. MCQ220, the question “Ever told you had cancer or malignancy?”.

This study was conducted in accordance with the Strengthening the Reporting of Cohort Studies in Surgery (STROCSS) guideline (23). Figure 1A depicts the 10 vitamin types and three modes of intake considered in this study. NHANES is a cross-sectional survey designed to gather a sample that represents non-institutionalized residents of the United States (24). We performed a cross-sectional analysis of male individuals aged 18 years and above within the combined group, utilizing data from six cycles spanning from 2007 to 2018, including information on vitamin intake and PCa status. Figure 1B presents a flowchart outlining the inclusion and exclusion criteria for study participants.

### 2.2 Data on vitamin intake and prostate cancer

The 24-h dietary recall method standed as the most widely employed approach for gathering dietary intake data at the national level, which serves to evaluate the consumption of various nutrient types (25, 26). During each NHANES cycle, participants provided detailed dietary intake data for two 24-h periods, which were subsequently utilized to estimate their vitamin intake (24, 27, 28). The first dietary recall was conducted in person during the visit, while the second recall was conducted via telephone 3 to 10 days afterward. For our analysis, the total estimated dietary vitamin intake (measured in mg or mcg) was averaged over the two recall periods. If data were available for only 1 day, the singular value was used for that participant. Participants with only one recall were retained in the analysis, and the two-day average was preferred if available. Additionally, participants were queried about their use of dietary supplements during the same two 24-h periods. Supplement-derived vitamin intake was similarly averaged over the 2 days when possible. Supplements included capsules, tablets, and other pill forms, were considered. Total vitamin intake was calculated as the sum of dietary and supplemental sources. Participants with missing information on supplement or total intake were excluded from the analysis. The distribution of vitamin intake is outlined in Supplementary Table S1. Cancer status was assessed through two consecutive questions: first, if the participant responded “Yes” to being asked “Have you ever been told you had cancer or malignancy?,” they were classified as cancer patients. Subsequently, participants were asked “What kind was it?,” allowing determination of the specific cancer type. Subjects who reported PCa were included in our study. Further details regarding this procedure can be found on the NHANES website.

### 2.3 Covariates

Demographic variables were obtained from demographic data in the NHANES database, encompassing age, race, body mass index (BMI), educational attainment, and the ratio of family income to poverty (PIR). Additionally, given that alcohol intake, smoking status, diabetes, and hypertension have been reported in previous literature to be associated with the risk of PCa, these variables were included as covariates in our analysis (29–32). Age was divided into three groups: 18–59 years, 60–79 years, and 80 years and older. Based on the 1997 guidelines established by the United States Office of Management and Budget, we divided the population into Mexican American, Non-Hispanic White, Non-Hispanic Black, Other Hispanic, and Other/multiracial. Body mass index (BMI) was obtained as weight (kg) divided by height (m^2^). Educational attainment was categorized as: Less Than 9th Grade, 9–11th Grade, High School Graduate or GED, Some College or Associate’s Degree, and College Graduate or above. Smoking status was categorized as: current smoker, former smoker, and never smoker. Alcohol consumption was categorized based on drinking frequency into non-drinkers, those who drink 1 to 5 times per month, 5 to 10 times per month, and more than 10 times per month. Diabetes status was determined based on a previous diagnosis by a physician. Specifically, participants who answered “yes” to the question, “Have you ever been told by a doctor or health professional that you have diabetes or sugar diabetes?” were classified into the diabetes group, while the others were assigned to the non-diabetes group. Hypertension was diagnosed if the participant answered “yes” to the question, “Have you ever been told by a doctor or other health professional that you had hypertension, also called high blood pressure?”.

### 2.4 Statistical analysis

Statistical analyses were conducted in accordance with the Centers for Disease Control and Prevention (CDC) analytical guidelines for complex NHANES survey data. Each participant was assigned a sample weight, and all analyses accounted for the complex survey design and weighting variables. Dietary and total vitamin intake were assessed using both continuous and categorical (quartile-based) approaches. To preserve the epidemiological and clinical interpretability of the odds ratios (ORs) and confidence intervals (CIs), we retained the original measurement units for each vitamin instead of converting them to a standardized unit. Categorical variables were analyzed using the chi-square test or Fisher’s exact test, while differences in continuous variables were assessed using the Kruskal–Wallis test. The proportion of missing covariate data was relatively low. Given that imputation could introduce uncertainty and additional assumptions, we opted not to impute the missing data. Weighted multivariable-adjusted logistic regression was used to calculate the OR and 95% CI for PCa risk across various continuous and categorical vitamin intake variables. Log-transformed supplements and total vitamin intake were used as exposure variables due to skewed distributions. A trend test was performed to evaluate the tendency between vitamin intake and PCa. We conducted an interaction analysis based on the group covariates. We also used restricted cubic spline (RCS) curves to evaluate the non-linear relationship. To ensure the robustness of the non-linear relationship, we performed a likelihood ratio test on the RCS model, conducted a concordance index (C-index) test to assess discrimination, and employed the Hosmer-Lemeshow Goodness-of-Fit test for calibration evaluation. We set the number of knots to four, used the median as the reference value, and performed non-linear tests of the model variables using the Wald method to calculate confidence intervals. In model diagnostics, the variance inflation factor (VIF) is a classical metric used to quantify the degree of linear correlation among explanatory variables and to identify potential multicollinearity. We calculated the VIFs for both the multivariable logistic regression model and the RCS model to assess the risk of multicollinearity and potential overfitting, thereby ensuring the interpretability and robustness of the diagnostic models. Statistical analyses were performed with R version 4.3.2. A *p*-value < 0.05 (two-tailed) was deemed statistically significant.

## 3 Results

### 3.1 Characteristics of participants

A total of 59,842 NHANES participants were surveyed in NHANES from 2007 to 2018. Male subjects aged 18 years or older were initially screened and 17,783 were obtained. After removing those with missing dietary and cancer essential information, 14,977 participants were finally included. Baseline characteristics of the participants are shown in Table 1, including 498 PCa patients (weighted to approximately 2.2%). Univariate analyses revealed statistically significant differences in age (*p* < 0.001), race (*p* < 0.001), education attainment (*p* = 0.009), PIR (*p* = 0.004), smoking status (*p* < 0.001), diabetes (*p* < 0.001) and hypertension (*p* < 0.001).

**Table 1:** Basic characteristics of participants among US adults in the cross-sectional study.

Characteristic	Overall, *N* = 14,977 (100%)	Prostate cancer		*P*-value
		No, *N* = 14,479 (98%)^2^	Yes, *N* = 498 (2.2%)	
Age				<0.001
18–59 years	9,578 (73%)	9,544 (74%)	34 (8.5%)	
60–79 years	4,140 (21%)	3,834 (20%)	306 (63%)	
80 + years	1,259 (6.2%)	1,101 (5.7%)	158 (29%)	
Race				<0.001
Mexican American	2,236 (9.2%)	2,211 (9.3%)	25 (2.1%)	
Other Hispanic	1,442 (5.7%)	1,410 (5.8%)	32 (2.8%)	
Non-Hispanic White	6,363 (66%)	6,102 (66%)	261 (75%)	
Non-Hispanic Black	3,185 (11%)	3,032 (11%)	153 (15%)	
Other/multiracial	1,751 (8.1%)	1,724 (8.2%)	27 (4.6%)	
BMI group				0.051
Normal (18.5 to <25)	3,850 (25%)	3,733 (25%)	117 (23%)	
Obesity (30 or greater)	5,209 (37%)	5,046 (37%)	163 (32%)	
Overweight (25 to <30)	5,565 (37%)	5,359 (37%)	206 (45%)	
Underweight (<18.5)	185 (1.1%)	179 (1.1%)	6 (0.7%)	
Drinking				0.4
1–5 drinks/month	6,504 (51%)	6,310 (51%)	194 (46%)	
10+ drinks/month	2,358 (24%)	2,276 (24%)	82 (25%)	
5–10 drinks/month	1,194 (12%)	1,163 (12%)	31 (11%)	
Non-drinker	1,994 (14%)	1,906 (14%)	88 (18%)	
Smoking				<0.001
Current smoker	3,560 (22%)	3,502 (22%)	58 (10.0%)	
Former smoker	4,515 (30%)	4,277 (29%)	238 (49%)	
Never smoker	6,891 (48%)	6,689 (48%)	202 (42%)	
Education attainment				0.009
Less Than 9th Grade	1,579 (5.3%)	1,528 (5.3%)	51 (5.3%)	
9-11th Grade	2,201 (11%)	2,139 (11%)	62 (9.6%)	
High School Grad/GED	3,603 (24%)	3,496 (24%)	107 (18%)	
Some College or AA degree	4,081 (30%)	3,950 (30%)	131 (28%)	
College Graduate or above	3,499 (29%)	3,352 (29%)	147 (39%)	
Ratio of family income to poverty	3.09 (1.51, 5.00)	3.09 (1.50, 5.00)	3.43 (1.98, 5.00)	0.004
BMI (kg/m^2^)	28.0 (24.8, 32.0)	28.0 (24.8, 32.1)	27.7 (25.2, 31.5)	0.7
Hypertension	5,422 (32%)	5,088 (32%)	334 (63%)	<0.001
Diabetes	2,123 (10%)	1,999 (10%)	124 (24%)	<0.001

Median (IQR) for continuous; *n* (%) for categorical. BMI, body mass index. GED, general educational development. AA, associate of arts.

### 3.2 Multivariate weighted logistic regression

We constructed three logistic regression models with progressive adjustment for covariates. Model 1 was unadjusted, while Model 2 adjusted for age, race, BMI, educational attainment, and PIR. Model 3 further incorporated adjustments for alcohol intake, smoking status, diabetes, and hypertension. Table 2 and Supplementary Tables S2–S4 outlines the multivariate weighted logistic regression results.

**Table 2:** Multivariate weighted logistic regression of the association between vitamin intake and prostate cancer among US adults in the cross-sectional study.

	Model 1			Model 2			Model 3		
	OR*^1^*	95% CI^1^	*p*	OR^1^	95% CI*^1^*	*p*	OR^1^	95% CI*^1^*	*p*
Vitamin A^#^									
Dietary vitamin intake	1.00	1.00, 1.00	**0.028**	1.00	1.00, 1.00	0.800	1.00	1.00, 1.00	>0.9
Vitamin B1^*^									
Dietary vitamin intake	0.95	0.81, 1.10	0.463	1.10	0.91, 1.34	0.329	0.99	0.80, 1.24	0.955
Supplement vitamin intake	0.82	0.66, 1.01	0.061	0.85	0.67, 1.07	0.171	0.82	0.64, 1.03	0.091
All vitamin intake	0.77	0.60, 1.00	**0.049**	0.85	0.66, 1.10	0.211	0.80	0.62, 1.03	0.086
Vitamin B2^*^									
Dietary vitamin intake	0.93	0.85, 1.01	0.075	0.98	0.86, 1.11	0.754	0.94	0.80, 1.09	0.402
Supplement vitamin intake	0.78	0.60, 1.01	0.059	0.81	0.61, 1.07	0.130	0.78	0.59, 1.04	0.087
All vitamin intake	0.71	0.52, 0.98	**0.037**	0.80	0.59, 1.09	0.151	0.78	0.58, 1.05	0.095
Vitamin B6^*^									
Dietary vitamin intake	0.89	0.82, 0.96	**0.004**	0.98	0.89, 1.07	0.633	0.89	0.78, 1.01	0.062
Supplement vitamin intake	0.85	0.67, 1.07	0.167	0.89	0.69, 1.14	0.346	0.87	0.65, 1.17	0.348
All vitamin intake	0.76	0.55, 1.05	0.095	0.86	0.63, 1.17	0.325	0.84	0.60, 1.19	0.319
Vitamin B9^#^									
Dietary vitamin intake	1.00	1.00, 1.00	**0.003**	1.00	1.00, 1.00	0.166	1.00	1.00, 1.00	0.106
Supplement vitamin intake	0.97	0.73, 1.28	0.818	0.75	0.53, 1.06	0.102	0.65	0.43, 1.00	**0.049**
All vitamin intake	0.57	0.34, 0.95	**0.033**	0.56	0.29, 1.08	0.084	0.50	0.22, 1.16	0.106
Vitamin B12^#^									
Dietary vitamin intake	0.99	0.97, 1.02	0.569	1.00	0.98, 1.02	0.866	0.99	0.97, 1.02	0.571
Supplement vitamin intake	1.02	0.89, 1.18	0.800	0.96	0.82, 1.12	0.600	0.83	0.70, 0.98	**0.033**
All vitamin intake	1.02	0.87, 1.20	0.771	0.96	0.81, 1.13	0.624	0.82	0.68, 0.99	**0.037**
Vitamin C^#^									
Dietary vitamin intake	1.00	1.00, 1.00	0.581	1.00	1.00, 1.00	0.886	1.00	1.00, 1.00	0.766
Supplement vitamin intake	1.02	0.87, 1.18	0.836	1.01	0.85, 1.21	0.887	0.94	0.78, 1.13	0.508
All vitamin intake	0.97	0.80, 1.18	0.777	0.99	0.77, 1.26	0.920	0.90	0.71, 1.15	0.389
Vitamin D^#^									
Dietary vitamin intake	1.02	0.99, 1.06	0.162	1.02	0.98, 1.06	0.276	0.99	0.96, 1.02	0.561
Supplement vitamin intake	1.34	1.09, 1.65	**0.006**	1.19	0.92, 1.55	0.179	1.09	0.85, 1.40	0.472
All vitamin intake	1.37	1.08, 1.75	**0.010**	1.22	0.91, 1.65	0.185	1.08	0.84, 1.40	0.550
Vitamin E^#^									
Dietary vitamin intake	0.99	0.97, 1.01	0.300	1.00	0.97, 1.02	0.800	1.00	0.97, 1.02	0.800
Vitamin K^*^									
Dietary vitamin intake	1.00	1.00, 1.00	0.674	1.00	1.00, 1.00	0.502	1.00	1.00, 1.00	0.563
Supplement vitamin intake	1.09	0.70, 1.69	0.716	1.17	0.70, 1.98	0.544	1.20	0.70, 2.06	0.494
All vitamin intake	0.93	0.69, 1.25	0.622	1.19	0.84, 1.68	0.318	1.12	0.74, 1.68	0.586

Model 1, no adjustment. Model 2, adjusted for age, race, BMI, education attainment, and ratio of family income to poverty. Model 3, adjusted for age, race, BMI, education attainment, ratio of family income to poverty, alcohol intake, smoking status, diabetes and hypertension. OR, odds ratio. CI, confidence interval. #, intake was calculated in mcg. *, intake was calculated in mg. The bold values in this table represent statistically significant results. The significance level is based on a p-value of less than 0.05 in the two-sided test.

**Figure 2:**
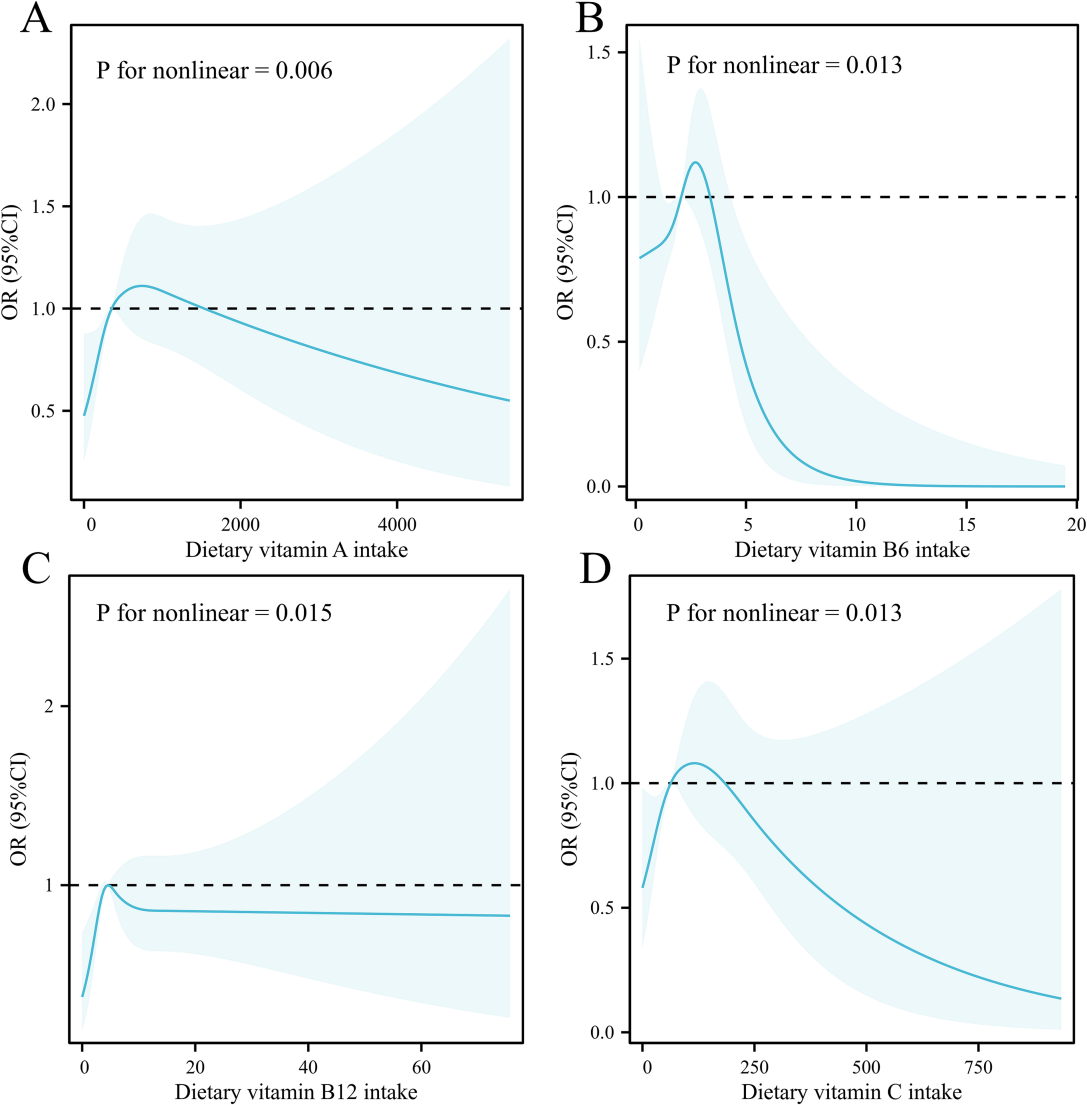
Restricted cubic splines for the association between **(A)** dietary vitamin A intake, **(B)** dietary vitamin B6 intake, **(C)** dietary vitamin B12 intake, and **(D)** dietary vitamin C intake and prostate cancer among US adults in the cross-sectional study.

**Figure 3:**
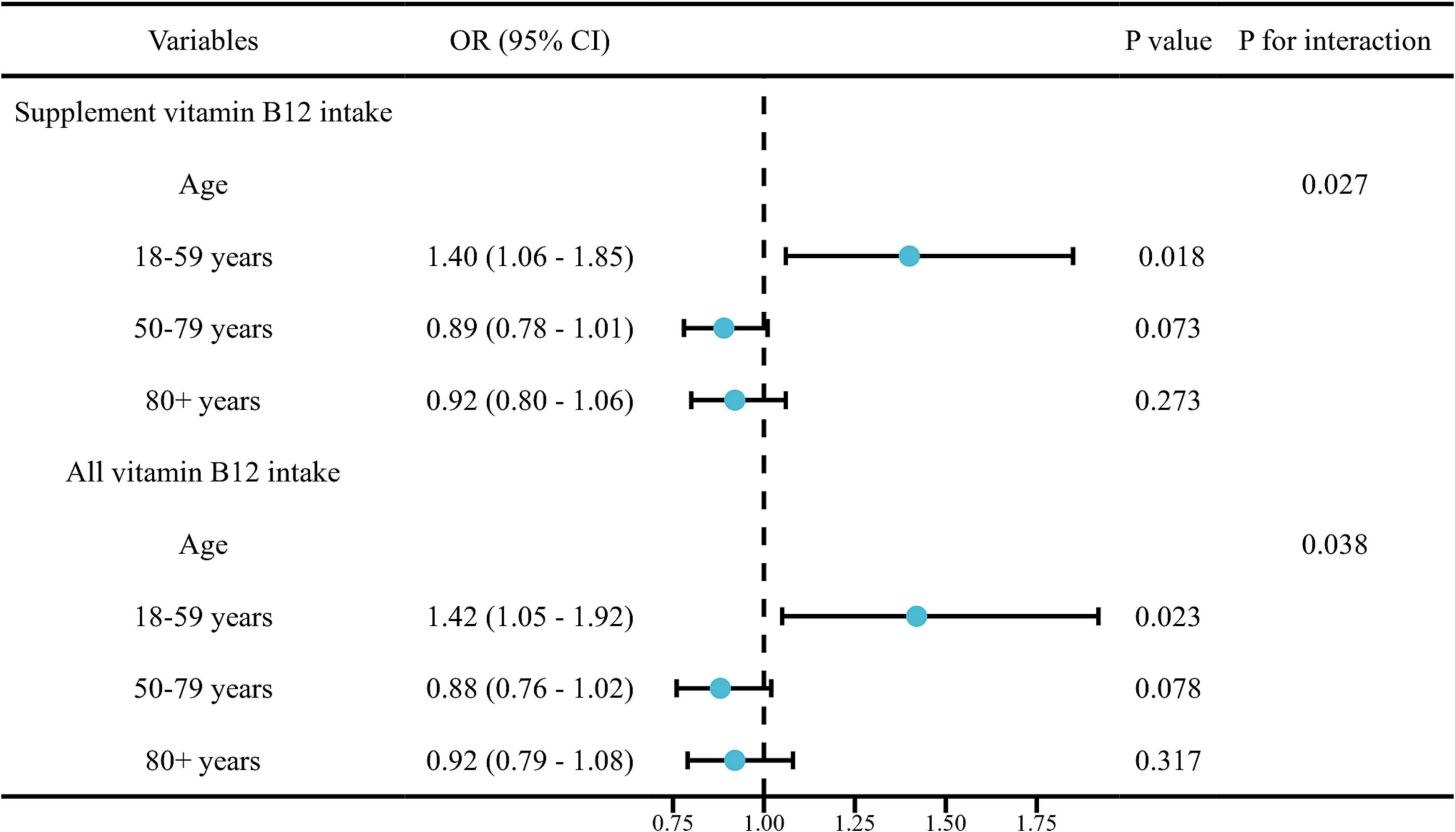
Interaction test for the association between dietary vitamin intake and prostate cancer among US adults in the cross-sectional study.

In the continuous variables analysis, Model 1 indicated a positive association between dietary retinol (vitamin A, mcg) intake (*p* = 0.028), supplement, and total vitamin D (mcg) intake (*p* = 0.006 for supplement; *p* = 0.010 for total) with PCa risk. Conversely, total vitamin B1 (mg) and B2 (mg) intake (*p* = 0.049 for B1; *p* = 0.037 for B2), dietary vitamin B6 (mg) intake (*p* = 0.004), and dietary and total vitamin B9 (mcg) intake (*p* = 0.003 for dietary; *p* = 0.033 for total) were negatively associated with PCa risk in Model 1. Following adjustments, the results revealed negative associations between supplement vitamin B9 (mcg) intake (OR = 0.65, 95% CI: 0.43, 1.00, *p* = 0.049), supplement vitamin B12 intake (mcg) (OR = 0.83, 95% CI: 0.70, 0.98, *p* = 0.030), and all vitamin B12 intake (mcg) (OR = 0.82, 95% CI: 0.68, 0.99, *p* = 0.037) with PCa risk. A 1.718-fold increase in supplemental vitamin B9, supplemental vitamin B12, and total vitamin B12 intake was associated with an approximately 35, 17, and 18% reduction in PCa risk, respectively. RCS curves showed significant non-linear relationships between dietary vitamin A (*p* = 0.006), vitamin B6 (*p* = 0.013), vitamin B12 (*p* = 0.015), vitamin C (*p* = 0.013), and PCa risk, respectively (Figure 2). RCS curves for the association between remaining different vitamin intakes and PCa can be found in Supplementary Figures S1–S3. The robustness analysis indicates that all models exhibit good fit and demonstrate strong accuracy. No evidence of multicollinearity was found (Supplementary Table S5). There is an interaction between supplementation and total vitamin B12 intake and age groups (Figure 3).

Regarding the analysis dividing vitamin intake into quartiles, a higher incidence of PCa was observed among individuals in the maximal quartile of dietary retinol intake (OR = 1.76, 95% CI = 1.07–2.88, *p* = 0.027, *p* for trend = 0.018) after adjustment. The unadjusted model showed positive associations between maximal quartile intakes of dietary vitamin C (OR = 1.54, 95% CI: 1.04, 2.27, *p* = 0.030), dietary vitamin K (OR = 1.38, 95% CI: 1.01, 1.90, *p* = 0.046), and total vitamin D (OR = 2.06, 95% CI: 1.04, 4.10, *p* = 0.039) with PCa risk. Additionally, total vitamins B1 (OR = 0.46, 95% CI: 0.24, 0.86, *p* = 0.016), B2 (OR = 0.40, 95% CI: 0.23, 0.73, *p* = 0.003), and B6 (OR = 0.40, 95% CI: 0.21, 0.75, *p* = 0.005) intake were negatively associated with PCa risk in the unadjusted model within the largest quartile of intake. However, these significant associations were not maintained after adjustment. In the fully adjusted model (Model 3), supplement intake of vitamin B1 (OR = 0.38; 95% CI: 0.16–0.94; *p* = 0.036) and vitamin B2 (OR = 0.35; 95% CI: 0.15–0.82; *p* = 0.016) was significantly associated with a reduced risk of PCa. A 1.718-fold increase in supplemental intake of vitamin B1 and vitamin B2 was associated with an approximately 62 and 65% reduction in PCa risk, respectively.

## 4 Discussion

This study is the first comprehensive investigation of the association between different types of vitamin intake and PCa risk based on large-scale observational study data. Specifically, we analyzed the association between the intake of 10 vitamins and PCa using NHANES data. The findings suggested that dietary retinol intake was positively associated with an increased risk of PCa. Additionally, there were inverse associations observed between supplemental vitamin B9 intake, supplemental vitamin B12 intake, and overall vitamin B12 intake with the risk of PCa.

Our cross-sectional study revealed an association between high dietary retinol intake and elevated PCa risk. Previous studies investigating the effect of retinol intake on PCa risk are limited and yield inconsistent results (14, 15). A case–control study conducted in Italy found no significant association between retinol and the risk of PCa (33). A study in southwestern Finland among adult male smokers found that higher serum retinol levels were associated with an increased risk of PCa (34). Our findings are also supported by a large prospective cohort study conducted in the United States involving more than 20,000 participants (35). Additionally, a case–control study in Europe reported a significant positive association between dietary retinol intake and an increased risk of PCa (36). Some studies on circulating retinol levels suggest that high concentrations of retinol may be beneficial in reducing PCa risk (37, 38). For instance, a large prospective study involving 30,000 men by Hada et al. (37) demonstrated higher levels of retinol were associated with an increased risk of PCa. Similarly, a recent case–control study in Singapore by Loh et al. (39) showed that increased retinol concentrations were positively associated with the overall risk of PCa. The potential mechanisms underlying the effect of retinol on PCa risk may include its contribution to tumorigenesis by promoting cell proliferation and dedifferentiation, thereby facilitating tumorigenesis (40). Given the potential regional differences, large-scale, multi-cohort studies are needed to further investigate these associations.

Our study found that supplemental intake of vitamins B1 and B2 was associated with a reduced risk of PCa. However, current epidemiological evidence regarding the relationship between these two vitamins and PCa is limited. Some studies have reported an inverse association between vitamin B1 intake and the risk of colorectal and esophageal cancers (41, 42). Additionally, a previous NHANES-based study identified a significant inverse correlation between vitamin B2 intake and prostate-specific antigen levels (43). Vitamin B1 may help maintain cellular homeostasis and reduce oxidative stress, while its deficiency could lead to DNA damage and impaired DNA repair capacity (44). Vitamin B2 may exert anti-PCa effects through its active form, flavin mononucleotide, which has been shown to compete with dihydrotestosterone for binding to the androgen receptor, thereby interfering with androgen signaling and exerting antitumor effects (45). Notably, the weak trends observed in our study suggest that the dose–response relationship may be non-linear or influenced by other factors. Therefore, we recommend future prospective cohort studies to improve the accuracy of exposure assessment, along with mechanistic studies to clarify the biological pathways involved. Additionally, future research should explore the effects of supplement form, dosage, and duration on PCa risk to provide more robust scientific evidence for nutritional strategies in PCa prevention. In the vitamin B family, our study revealed that vitamin B9 and B12 intake were negatively associated with PCa risk. Several studies have suggested that folic acid, a component of vitamin B9, may protect effects against PCa through mechanisms such as cytosine-phosphate islands, DNA uracil misincorporation, and methylation (46). Similarly, a case–control study by Shannon et al. (11) discovered a negative association between dietary folate intake and PCa risk, and other studies have suggest that higher folate levels may protect against elevated prostate-specific antigen levels (47). However, our study only showed a significant protective effect of vitamin B9 supplementation in PCa risk. The differences in bioavailability between supplemental and dietary vitamin B9 may help explain their varying associations with PCa risk. A randomized controlled trial (RCT) in lactating women found that 96% of those taking 400 μg/day of synthetic folic acid had detectable levels of unmetabolized folic acid (UMFA) in their breast milk, accounting for approximately 8% of the total milk folate concentration (48). In contrast, this phenomenon was not observed in women supplemented with the natural form, [6S]-5-methyltetrahydrofolate (5-methylTHF) (48). These findings suggest that the metabolic capacity for synthetic folic acid in the intestine and liver can be saturated, potentially leading to UMFA accumulation. Such accumulation may disrupt the body’s folate regulation mechanisms and downstream functions, such as folate-binding protein expression, ultimately affecting folate bioavailability in specific tissues. Additionally, dietary intake was self-reported by participants, which may introduce recall bias. Tio et al. (12) conducted a meta-analysis of 11 studies with 146,782 participants and found no association between dietary vitamin B9 and PCa risk. Similar findings were reported in a meta-analysis by Wang et al. (49), which aligns with our study results. Furthermore, our cross-sectional investigation revealed a negative correlation between supplemental vitamin B12 consumption and PCa risk. However, there is a lack of studies on the association between vitamin B12 intake and PCa risk. Although several studies have examined the link between circulating levels of vitamin B12 and the risk of PCa, their findings have been inconsistent (10, 13). It is hypothesized that vitamin B may influence cancer risk through its effects on DNA replication, methylation, and cellular damage repair (50). The intake of other B vitamins is not associated with PCa risk observed in population-based studies. Considering that cross-sectional studies are limited in their ability to establish causality, further large prospective population-based trials or RCTs are warranted to address the existing gap in this area.

Our study found no association between the intake of vitamin C or vitamin D and the risk of PCa. Vitamin C, known for its role as a reducing agent and scavenger of free radicals, has been suggested to have potential implications in anticancer treatment. However, two randomized trials found no link between vitamin C dietary intake or supplementation and PCa risk (51, 52). Similarly, a case–control study in Canada involving approximately 4,000 participants found no association between dietary intake or supplementation of vitamin C and the incidence of PCa (53). Vitamin D, which primarily regulates calcium and phosphorus metabolism, has been suggested to influence tumor development by regulating cell differentiation and apoptosis (54). Nevertheless, no association between vitamin D intake and PCa risk was found in this study. This aligns with recent systematic reviews and a large RCT (7, 55). It is important to note that dietary intake alone is not the only factor influencing vitamin D levels in the blood, and thus, the relationship between vitamin D intake and PCa risk warrants further exploration.

Vitamin E has shown antitumor activity in some preclinical studies due to its pro-apoptotic properties, but this has not been consistently observed in population studies (19, 51, 56). Similarly, our observational study found no association between vitamin E and PCa risk. Vitamin K is available in two natural forms, phylloquinone (vitamin K-1) and menaquinones (vitamin K-2), with the former serving as the primary dietary form derived from vegetable oils and vegetables (57). Preclinical evidence suggests that vitamin K may exert antitumor activity, as demonstrated in several *in vitro* and *in vivo* studies. For example, a prospective cohort study by Nimptsch et al. (58) discovered that menaquinones intake showed an inverse association with the risk of PCa (21). However, some studies have proposed that vitamin K antagonists may have antitumor properties, though these findings were not confirmed in a subsequent nested case–control study using the Danish Demographic and Health Data Register (22, 59, 60). In our study, no association between vitamin K intake and PCa risk was observed. The outcomes of a large cancer screening trial in the United States population conducted by Hoyt et al. indicated that intake of vitamin K did not affect the overall and advanced PCa risk in the US population (61). Mechanistically, vitamin K exerts its antitumor effects by enhancing oxidative stress and causing cell cycle arrest, while vitamin K antagonists inhibit peroxisome proliferator-activated receptor *γ* signaling, subsequently inhibiting AR signaling to suppress PCa (21, 62). Thus, the relationship between vitamin K and PCa remains elusive.

Our study exhibits several strengths that contribute to its reliability. Firstly, we employed a comprehensive set of factors as covariates in our observational study to minimize confounding effects, which strengthens the robustness of our results. Secondly, we utilized a nationally representative NHANES population, thereby ensuring the generalizability and statistical power of our analyses. However, our study also has limitations. Firstly, the methodological approaches correspond to populations from a multiracial US population, potentially limiting the generalizability of our findings. Secondly, certain vitamin species were excluded from the analysis due to data limitations. Furthermore, the design of this study limits our ability to infer the direction of causality between vitamin intake and PCa risk, raising the possibility of reverse causation. For instance, individuals may change their vitamin intake after being diagnosed with PCa or due to the presence of other health conditions. The inconsistency of results across models adjusted for different sets of covariates suggests that the findings should be interpreted with caution. Although the 24-h dietary recall is widely used in nutritional epidemiology, its reliance on self-reported data may introduce recall bias, potentially attenuating true associations. Confounding by indication may be present among supplement users, as individuals who choose to take supplements may differ systematically in health status or behavior compared to non-users. The absence of a clearly defined strategy for multiple comparisons increases the risk of type I error, which may lead to spurious associations. The sample size of PCa patients in this study is relatively small, which may affect statistical power and lead to result instability and potential bias. Although we have adjusted for several covariates, there may still be residual confounding factors that could impact the results. We observed that in the quartile-based analysis, the associations between certain vitamin intakes and the risk of PCa were not entirely consistent with the corresponding trend tests. In addition to factors such as sample size and potential confounding, this discrepancy may be attributed to limitations inherent in the statistical methods as well as the complex underlying biological mechanisms of vitamin metabolism. Since the data were log-transformed, the clinical interpretation of the results should be understood on the logarithmic scale. We recommend that future studies further consider standardizing measurement units during data processing to enhance interpretability and consistency of the findings. Considering that the dose–response relationship may exhibit more complex non-linear patterns, future studies should employ more sophisticated analytical methods, such as polynomial regression or piecewise regression, to more comprehensively elucidate deeper non-linear effects. Third, PCa status in our study was based on self-reported data, which is subject to potential recall bias and outcome misclassification. Participants may inaccurately recall or report their cancer diagnosis, particularly for diseases with long latency such as PCa. Misclassification of disease status could lead to underestimation or overestimation of true associations. Over-reporting among health-conscious individuals with higher supplement use might lead to an underestimation of the protective effect of vitamin B12, while under-reporting in individuals with high dietary retinol intake could exaggerate its apparent risk association. Due to methodological constraints, we did not evaluate the potential correlations among the intake of multiple vitamins. Future research should establish standardized metrics for vitamin intake and utilize prospective cohort studies and RCTs to comprehensively assess the combined effects of multiple vitamins.

## 5 Conclusion

In conclusion, our observational study suggests that dietary intake of retinol may increase the PCa risk, while supplemental intake of vitamin B9, vitamin B12, and total vitamin B12 intake are inversely associated with PCa risk. However, no significant associations were found between the intake of other vitamins and PCa risk. Due to the study design, we cannot establish definitive causality between vitamin intake and PCa risk. Future research should focus on high-quality, large-scale RCTs to strengthen the evidence for vitamin intake as a preventive measure for PCa.

## Data Availability

The original contributions presented in the study are included in the article/Supplementary material, further inquiries can be directed to the corresponding author.
