# Supplementary material for: Association between vitamin intake and prostate cancer: a cross-sectional study
Source: Front Nutr. 2025 Jun 13;12:1607452. doi: 10.3389/fnut.2025.1607452 (PMC12202377; doi:10.3389/fnut.2025.1607452)
Supplement: Supplementary file 1 [file Table_1.docx]

Supplementary Material


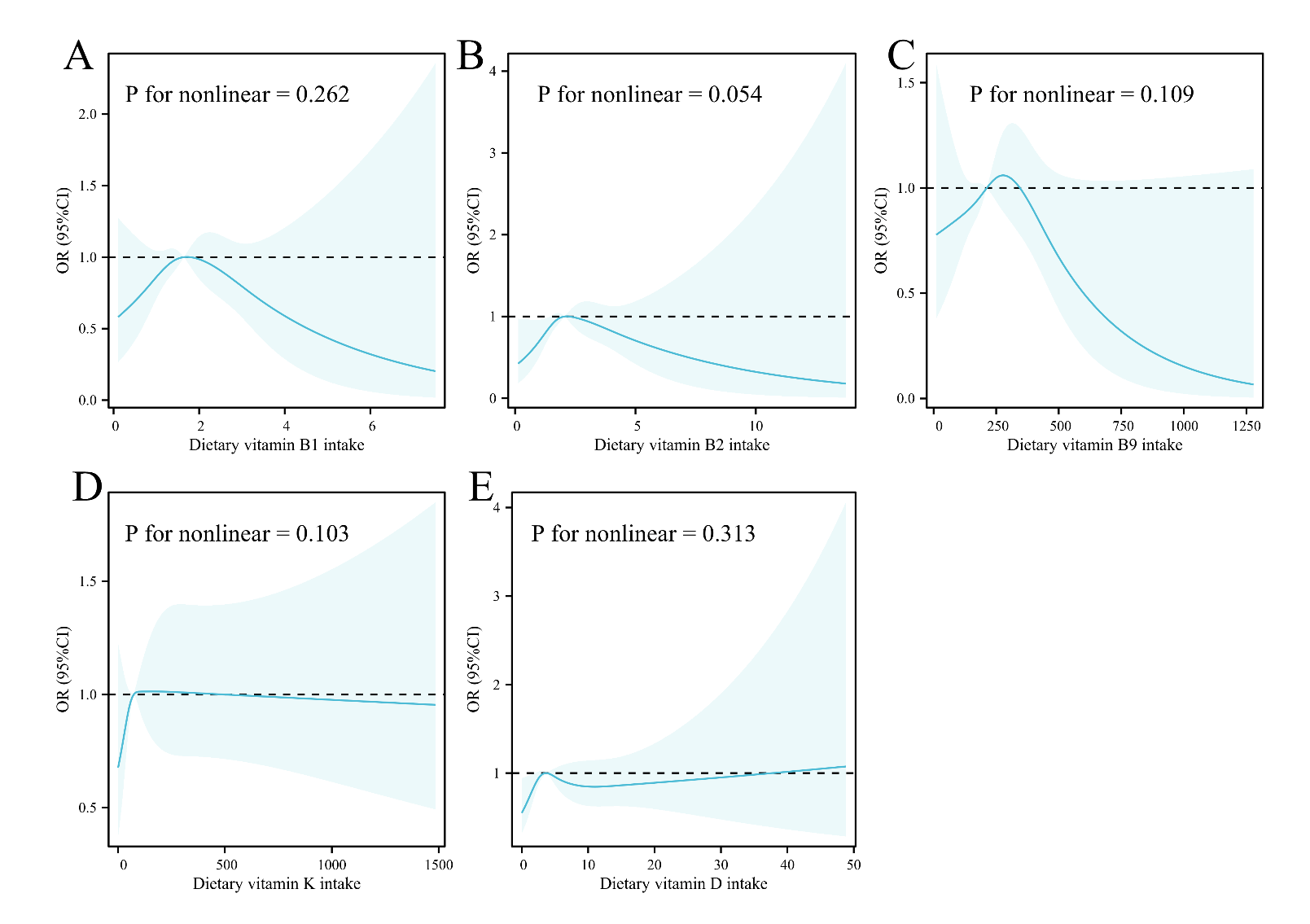


Supplementary Figure S1. Restricted cubic splines for the association between dietary (A) vitamin B1, (B) vitamin B2, (C) vitamin B9, (D) vitamin K, and (E) vitamin D intake and prostate cancer among US adults in the cross-sectional study (p for nonlinear > 0.05).


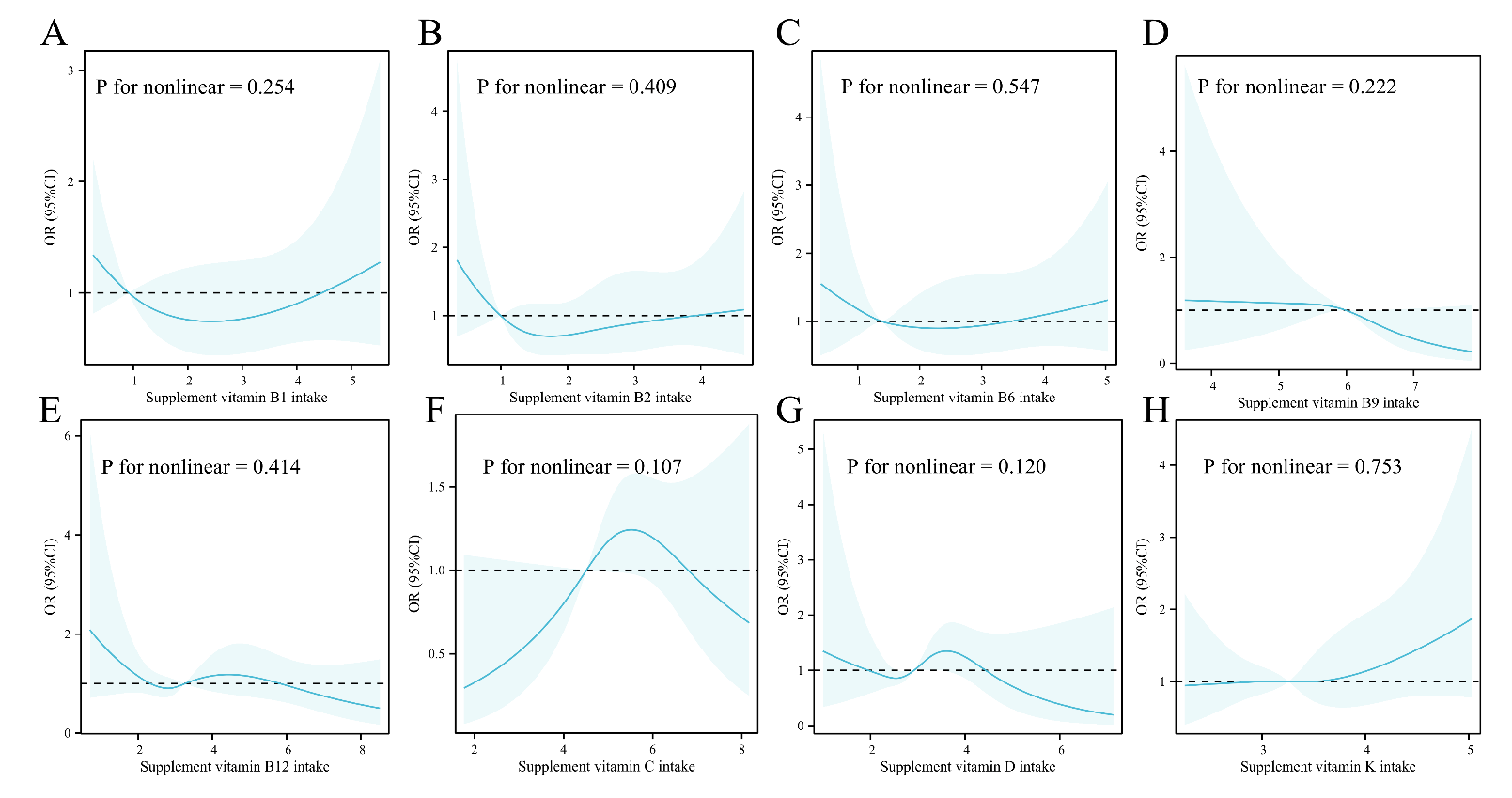


Supplementary Figure S2. Restricted cubic splines for the association between supplement (A) vitamin B1, (B) vitamin B2, (C) vitamin B6, (D) vitamin B9, (E) vitamin B12, (F) vitamin C, (G) vitamin D, and (H) vitamin K intake and prostate cancer among US adults in the cross-sectional study.


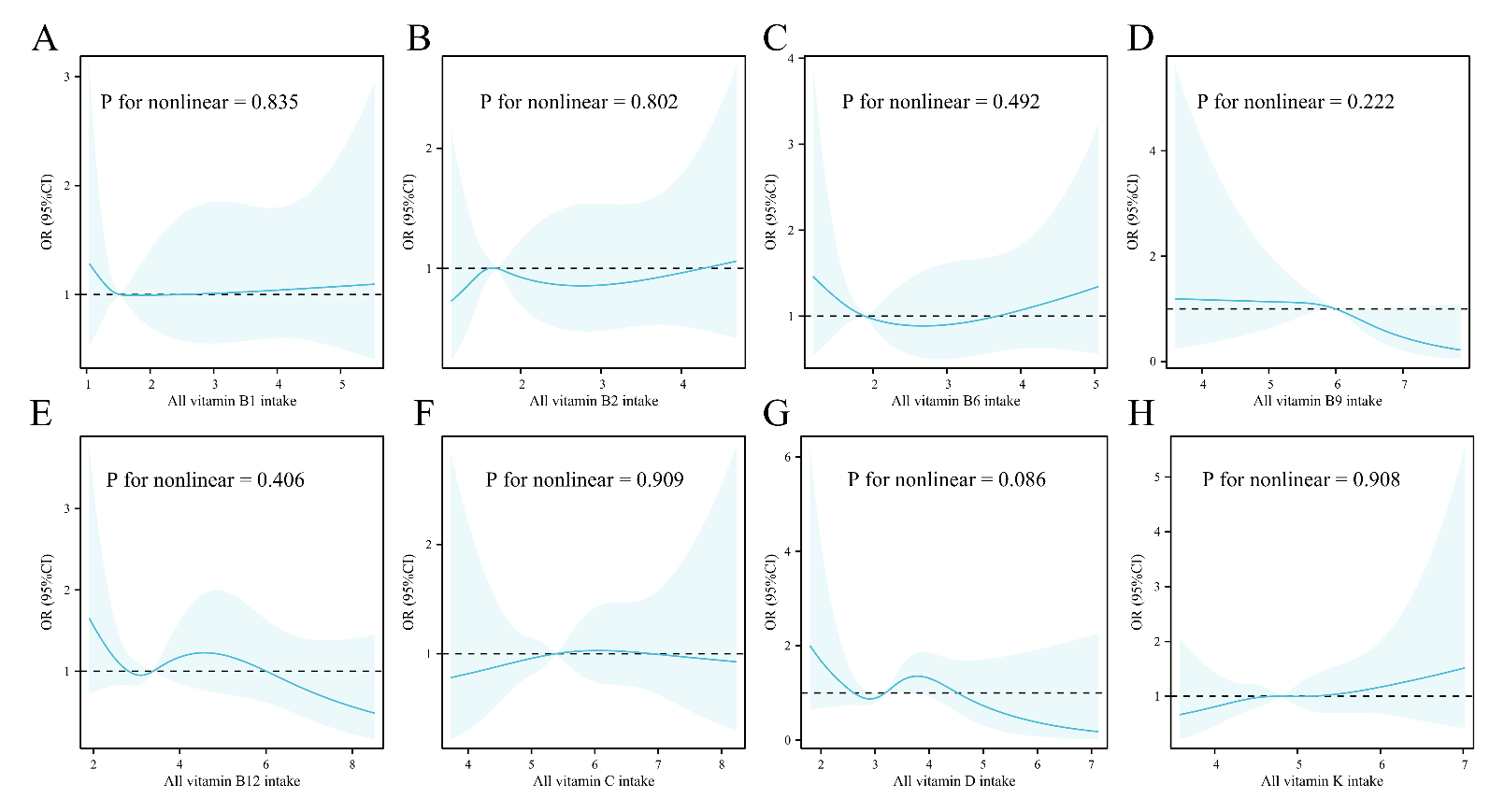


Supplementary Figure S3. Restricted cubic splines for the association between all (A) vitamin B1, (B) vitamin B2, (C) vitamin B6, (D) vitamin B9, (E) vitamin B12, (F) vitamin C, (G) vitamin D, and (H) vitamin K vitamin intake and prostate cancer among US adults in the cross-sectional study.

Supplementary Table S1. Quartile classification of vitamin intake.

| Dietary intake type | Unit | Q1 | Q2 | Q3 | Q4 |
| --- | --- | --- | --- | --- | --- |
| Vitamin A | mcg | 70 (19, 128) | 263 (217, 305) | 450 (399, 507) | 796 (666, 1,005) |
| Vitamin B1 | mg | 1.02 (0.85, 1.16) | 1.49 (1.38, 1.61) | 1.97 (1.84, 2.11) | 2.79 (2.49, 3.32) |
| Vitamin B2 | mg | 1.28 (1.04, 1.46) | 1.94 (1.79, 2.07) | 2.54 (2.38, 2.74) | 3.67 (3.27, 4.43) |
| Vitamin B6 | mg | 1.22 (0.98, 1.42) | 1.88 (1.73, 2.03) | 2.53 (2.36, 2.74) | 3.77 (3.28, 4.83) |
| Vitamin B9 | mcg | 123 (96, 141) | 190 (175, 206) | 259 (240, 279) | 377 (333, 454) |
| Vitamin B12 | mcg | 2.2 (1.5, 2.7) | 4.0 (3.5, 4.4) | 6.0 (5.4, 6.6) | 10.1 (8.4, 13.1) |
| Vitamin C | mg | 16 (10, 22) | 44 (36, 52) | 87 (74, 102) | 173 (141, 228) |
| Vitamin D | mcg | 1.1 (0.6, 1.5) | 2.9 (2.5, 3.4) | 5.1 (4.5, 5.8) | 9.9 (7.9, 13.6) |
| Vitamin E | mg | 4.2 (3.3, 4.9) | 6.8 (6.2, 7.5) | 9.6 (8.8, 10.5) | 15.4 (13.3, 19.5) |
| Vitamin K | mg | 34 (24, 41) | 62 (55, 70) | 103 (90, 117) | 201 (159, 286) |
| Supplement intake type | Unit* | Q1 | Q2 | Q3 | Q4 |
| Vitamin B1 | mg | 0.92 (0.85, 0.92) | 1.79 (1.70, 2.15) | 3.27 (3.26, 3.93) | 4.62 (3.96, 4.63) |
| Vitamin B2 | mg | 0.99 (0.99, 0.99) | 1.79 (1.48, 2.21) | 3.12 (3.04, 3.26) | 3.93 (3.93, 4.11) |
| Vitamin B6 | mg | 1.39 (1.10, 1.39) | 2.40 (2.20, 2.77) | 3.71 (3.26, 3.93) | 4.62 (4.56, 4.64) |
| Vitamin B9 | mcg | 5.30 (4.95, 5.30) | 5.99 (5.99, 5.99) | 6.69 (6.40, 6.69) | 7.00 (6.91, 7.22) |
| Vitamin B12 | mcg | 18 (6, 25) | 56 (50, 100) | 506 (250, 1,000) | 1,506 (1,025, 2,500) |
| Vitamin C | mg | 4.11 (4.11, 4.51) | 5.35 (5.02, 5.71) | 6.33 (6.22, 6.38) | 6.99 (6.91, 7.34) |
| Vitamin D | mcg | 2.40 (2.40, 2.44) | 3.04 (2.92, 3.26) | 3.93 (3.71, 4.11) | 4.93 (4.84, 5.30) |
| Vitamin K | mg | 3.04 (3.04, 3.04) | 3.26 (3.26, 3.43) | 4.11 (4.11, 4.39) | 4.68 (4.62, 4.80) |
| All intake type | Unit* | Q1 | Q2 | Q3 | Q4 |
| Vitamin B1 | mg | 1.29 (1.23, 1.33) | 1.46 (1.42, 1.50) | 1.69 (1.62, 1.82) | 3.53 (2.97, 4.00) |
| Vitamin B2 | mg | 1.44 (1.36, 1.48) | 1.62 (1.58, 1.67) | 1.84 (1.78, 1.94) | 3.29 (2.68, 3.96) |
| Vitamin B6 | mg | 1.58 (1.48, 1.64) | 1.80 (1.75, 1.85) | 2.10 (1.98, 2.21) | 3.37 (2.76, 4.00) |
| Vitamin B9 | mcg | 6.21 (6.05, 6.27) | 6.45 (6.39, 6.52) | 6.69 (6.63, 6.75) | 7.01 (6.93, 7.18) |
| Vitamin B12 | mcg | 2.49 (2.32, 2.67) | 3.22 (3.10, 3.34) | 3.54 (3.46, 3.89) | 6.22 (4.70, 6.92) |
| Vitamin C | mg | 4.64 (4.43, 4.83) | 5.22 (5.09, 5.34) | 5.80 (5.62, 6.06) | 6.80 (6.48, 7.10) |
| Vitamin D | mcg | 2.78 (2.61, 2.93) | 3.33 (3.21, 3.44) | 3.94 (3.77, 3.99) | 4.69 (4.37, 4.96) |
| Vitamin K | mg | 4.22 (4.05, 4.38) | 4.68 (4.57, 4.79) | 5.11 (5.00, 5.21) | 5.65 (5.47, 6.08) |

*Supplement and all intake both performed a log(n+1) transformation.

Supplementary Table S2. Multivariate weighted logistic regression of the association between dietary vitamin intake quartiles and prostate cancer among US adults in the cross-sectional study.

| Dietary vitamin intake type | Model 1 | | | Model 2 | | | Model 3 | | |
| --- | --- | --- | --- | --- | --- | --- | --- | --- | --- |
|  | OR*^1^* | 95% CI*^1^* | p | OR*^1^* | 95% CI*^1^* | p | OR*^1^* | 95% CI*^1^* | p |
| Vitamin A |  |  |  |  |  |  |  |  |  |
| Q1 | Ref | — | — | Ref | — | — | Ref | — | — |
| Q2 | 1.58 | 1.03, 2.43 | 0.036 | 1.27 | 0.83, 1.96 | 0.300 | 1.44 | 0.89, 2.34 | 0.140 |
| Q3 | 2.05 | 1.46, 2.87 | <0.001 | 1.41 | 0.98, 2.01 | 0.062 | 1.65 | 1.08, 2.52 | 0.022 |
| Q4 | 2.12 | 1.43, 3.13 | <0.001 | 1.60 | 1.03, 2.47 | 0.037 | 1.76 | 1.07, 2.88 | 0.027 |
| p for trend |  |  | <0.001 |  |  | 0.027 |  |  | 0.018 |
| Vitamin B1 |  |  |  |  |  |  |  |  |  |
| Q1 | Ref | — | — | Ref | — | — | Ref | — | — |
| Q2 | 1.43 | 1.04, 1.97 | 0.030 | 1.48 | 1.01, 2.15 | 0.043 | 1.76 | 1.14, 2.71 | 0.011 |
| Q3 | 1.07 | 0.72, 1.60 | 0.700 | 1.25 | 0.79, 1.99 | 0.300 | 1.29 | 0.78, 2.13 | 0.300 |
| Q4 | 1.10 | 0.79, 1.55 | 0.600 | 1.49 | 0.99, 2.24 | 0.058 | 1.32 | 0.85, 2.04 | 0.200 |
| p for trend |  |  | 0.985 |  |  | 0.133 |  |  | 0.472 |
| Vitamin B2 |  |  |  |  |  |  |  |  |  |
| Q1 | Ref | — | — | Ref | — | — | Ref | — | — |
| Q2 | 1.03 | 0.73, 1.46 | 0.900 | 0.90 | 0.61, 1.33 | 0.600 | 0.94 | 0.60, 1.47 | 0.800 |
| Q3 | 1.14 | 0.85, 1.52 | 0.400 | 1.06 | 0.73, 1.53 | 0.800 | 0.90 | 0.59, 1.38 | 0.600 |
| Q4 | 0.86 | 0.58, 1.29 | 0.500 | 0.92 | 0.57, 1.49 | 0.700 | 0.82 | 0.49, 1.40 | 0.500 |
| p for trend |  |  | 0.559 |  |  | 0.909 |  |  | 0.436 |
| Vitamin B6 |  |  |  |  |  |  |  |  |  |
| Q1 | Ref | — | — | Ref | — | — | Ref | — | — |
| Q2 | 0.92 | 0.68, 1.26 | 0.600 | 0.98 | 0.68, 1.40 | 0.900 | 1.01 | 0.67, 1.51 | >0.9 |
| Q3 | 0.87 | 0.57, 1.34 | 0.500 | 1.05 | 0.64, 1.72 | 0.800 | 1.16 | 0.70, 1.93 | 0.600 |
| Q4 | 0.79 | 0.55, 1.13 | 0.200 | 1.12 | 0.72, 1.76 | 0.600 | 0.90 | 0.56, 1.47 | 0.700 |
| p for trend |  |  | 0.194 |  |  | 0.564 |  |  | 0.836 |
| Vitamin B9 |  |  |  |  |  |  |  |  |  |
| Q1 | Ref | — | — | Ref | — | — | Ref | — | — |
| Q2 | 1.28 | 0.92, 1.77 | 0.140 | 1.30 | 0.92, 1.84 | 0.140 | 1.32 | 0.92, 1.88 | 0.130 |
| Q3 | 0.92 | 0.64, 1.32 | 0.600 | 1.05 | 0.70, 1.57 | 0.800 | 0.83 | 0.53, 1.29 | 0.400 |
| Q4 | 0.82 | 0.57, 1.16 | 0.300 | 0.98 | 0.63, 1.53 | >0.9 | 0.88 | 0.55, 1.43 | 0.600 |
| p for trend |  |  | 0.106 |  |  | 0.700 |  |  | 0.307 |
| Vitamin B12 |  |  |  |  |  |  |  |  |  |
| Q1 | Ref | — | — | Ref | — | — | Ref | — | — |
| Q2 | 1.40 | 0.99, 1.97 | 0.056 | 1.57 | 1.08, 2.29 | 0.019 | 1.71 | 1.13, 2.60 | 0.012 |
| Q3 | 1.05 | 0.70, 1.58 | 0.800 | 1.08 | 0.67, 1.72 | 0.800 | 1.05 | 0.63, 1.75 | 0.900 |
| Q4 | 1.04 | 0.73, 1.48 | 0.800 | 1.32 | 0.87, 1.99 | 0.200 | 1.18 | 0.75, 1.86 | 0.500 |
| p for trend |  |  | 0.765 |  |  | 0.512 |  |  | 0.988 |
| Vitamin C |  |  |  |  |  |  |  |  |  |
| Q1 | Ref | — | — | Ref | — | — | Ref | — | — |
| Q2 | 1.73 | 1.10, 2.71 | 0.018 | 1.63 | 1.00, 2.67 | 0.050 | 1.16 | 0.69, 1.97 | 0.600 |
| Q3 | 2.07 | 1.39, 3.06 | <0.001 | 1.68 | 1.06, 2.67 | 0.028 | 1.39 | 0.86, 2.26 | 0.200 |
| Q4 | 1.54 | 1.04, 2.27 | 0.030 | 1.37 | 0.85, 2.21 | 0.200 | 1.13 | 0.67, 1.89 | 0.600 |
| p for trend |  |  | 0.010 |  |  | 0.173 |  |  | 0.492 |
| Vitamin D |  |  |  |  |  |  |  |  |  |
| Q1 | Ref | — | — | Ref | — | — | Ref | — | — |
| Q2 | 1.45 | 0.97, 2.17 | 0.071 | 1.11 | 0.69, 1.76 | 0.700 | 1.29 | 0.76, 2.20 | 0.300 |
| Q3 | 1.93 | 1.32, 2.82 | <0.001 | 1.42 | 0.92, 2.18 | 0.110 | 1.69 | 1.05, 2.71 | 0.030 |
| Q4 | 1.37 | 0.94, 2.00 | 0.100 | 1.01 | 0.66, 1.57 | >0.9 | 0.97 | 0.60, 1.57 | >0.9 |
| p for trend |  |  | 0.044 |  |  | 0.676 |  |  | 0.808 |
| Vitamin E |  |  |  |  |  |  |  |  |  |
| Q1 | Ref | — | — | Ref | — | — | Ref | — | — |
| Q2 | 0.81 | 0.57, 1.14 | 0.200 | 0.75 | 0.49, 1.13 | 0.200 | 0.82 | 0.52, 1.29 | 0.400 |
| Q3 | 0.86 | 0.63, 1.18 | 0.300 | 0.91 | 0.63, 1.34 | 0.600 | 1.02 | 0.66, 1.59 | >0.9 |
| Q4 | 0.96 | 0.69, 1.34 | 0.800 | 1.03 | 0.65, 1.63 | >0.9 | 1.01 | 0.61, 1.69 | >0.9 |
| p for trend |  |  | 0.917 |  |  | 0.679 |  |  | 0.739 |
| Vitamin K |  |  |  |  |  |  |  |  |  |
| Q1 | Ref | — | — | Ref | — | — | Ref | — | — |
| Q2 | 1.51 | 1.08, 2.13 | 0.018 | 1.45 | 0.99, 2.13 | 0.056 | 1.49 | 0.97, 2.30 | 0.069 |
| Q3 | 1.43 | 1.03, 1.99 | 0.033 | 1.21 | 0.81, 1.80 | 0.300 | 1.08 | 0.66, 1.77 | 0.800 |
| Q4 | 1.38 | 1.01, 1.90 | 0.046 | 1.16 | 0.78, 1.72 | 0.500 | 1.15 | 0.73, 1.80 | 0.500 |
| p for trend |  |  | 0.060 |  |  | 0.668 |  |  | 0.894 |

Model 1, no adjustment. Model 2, adjusted for age, race, BMI, education attainment, and ratio of family income to poverty. Model 3, adjusted for age, race, BMI, education attainment, ratio of family income to poverty, alcohol intake, smoking status, diabetes and hypertension. OR, odds ratio. CI, confidence interval.

Supplementary Table S3. Multivariate weighted logistic regression of the association between supplement vitamin intake quartiles and prostate cancer among US adults in the cross-sectional study.

| Supplement vitamin intake type | Model 1 | | | Model 2 | | | Model 3 | | |
| --- | --- | --- | --- | --- | --- | --- | --- | --- | --- |
|  | OR*^1^* | 95% CI*^1^* | p | OR*^1^* | 95% CI*^1^* | p | OR*^1^* | 95% CI*^1^* | p |
| Vitamin B1 |  |  |  |  | Q1 | — | — |  |  |
| Q1 | Ref | — | — | Ref | — | — | Ref | — | — |
| Q2 | 0.52 | 0.22, 1.22 | 0.130 | 0.59 | 0.24, 1.47 | 0.300 | 0.84 | 0.34, 2.06 | 0.700 |
| Q3 | 0.65 | 0.32, 1.36 | 0.300 | 0.80 | 0.34, 1.88 | 0.600 | 0.82 | 0.32, 2.12 | 0.700 |
| Q4 | 0.44 | 0.22, 0.89 | 0.023 | 0.52 | 0.24, 1.12 | 0.092 | 0.38 | 0.16, 0.94 | 0.036 |
| p for trend |  |  | 0.084 |  |  | 0.263 |  |  | 0.080 |
| Vitamin B2 |  |  |  |  |  |  |  |  |  |
| Q1 | Ref | — | — | Ref | — | — | Ref | — | — |
| Q2 | 0.44 | 0.20, 0.98 | 0.043 | 0.49 | 0.21, 1.16 | 0.100 | 0.65 | 0.28, 1.54 | 0.300 |
| Q3 | 0.53 | 0.21, 1.32 | 0.200 | 0.81 | 0.33, 1.99 | 0.600 | 0.85 | 0.30, 2.38 | 0.800 |
| Q4 | 0.58 | 0.28, 1.21 | 0.140 | 0.49 | 0.20, 1.21 | 0.120 | 0.35 | 0.15, 0.82 | 0.016 |
| p for trend |  |  | 0.283 |  |  | 0.323 |  |  | 0.323 |
| Vitamin B6 |  |  |  |  |  |  |  |  |  |
| Q1 | Ref | — | — | Ref | — | — | Ref | — | — |
| Q2 | 0.31 | 0.11, 0.90 | 0.032 | 0.48 | 0.16, 1.40 | 0.200 | 0.60 | 0.20, 1.84 | 0.400 |
| Q3 | 0.59 | 0.28, 1.23 | 0.200 | 0.70 | 0.30, 1.67 | 0.400 | 0.89 | 0.34, 2.35 | 0.800 |
| Q4 | 0.83 | 0.32, 2.16 | 0.700 | 0.85 | 0.29, 2.47 | 0.800 | 0.75 | 0.21, 2.69 | 0.700 |
| p for trend |  |  | 0.971 |  |  | 0.943 |  |  | 0.823 |
| Vitamin B9 |  |  |  |  |  |  |  |  |  |
| Q1 | Ref | — | — | Ref | — | — | Ref | — | — |
| Q2 | 1.36 | 0.57, 3.20 | 0.500 | 0.75 | 0.31, 1.82 | 0.500 | 0.75 | 0.31, 1.82 | 0.500 |
| Q3 | 0.66 | 0.19, 2.24 | 0.500 | 0.40 | 0.12, 1.38 | 0.150 | 0.40 | 0.12, 1.38 | 0.150 |
| Q4 | 1.48 | 0.51, 4.28 | 0.500 | 0.50 | 0.12, 2.04 | 0.300 | 0.50 | 0.12, 2.04 | 0.300 |
| p for trend |  |  | 0.790 |  |  | 0.210 |  |  | 0.225 |
| Vitamin B12 |  |  |  |  |  |  |  |  |  |
| Q1 | — | — |  | — | — |  | — | — |  |
| Q2 | 1.19 | 0.63, 2.24 | 0.600 | 1.13 | 0.54, 2.35 | 0.700 | 0.87 | 0.45, 1.70 | 0.700 |
| Q3 | 1.10 | 0.50, 2.44 | 0.800 | 0.98 | 0.42, 2.30 | >0.9 | 0.41 | 0.20, 0.86 | 0.018 |
| Q4 | 1.13 | 0.54, 2.38 | 0.700 | 0.78 | 0.34, 1.79 | 0.600 | 0.64 | 0.22, 1.87 | 0.400 |
| p for trend |  |  | 0.808 |  |  | 0.497 |  |  | 0.214 |
| Vitamin C |  |  |  |  |  |  |  |  |  |
| Q1 | Ref | — | — | Ref | — | — | Ref | — | — |
| Q2 | 1.20 | 0.74, 1.92 | 0.500 | 1.20 | 0.71, 2.04 | 0.500 | 1.14 | 0.64, 2.01 | 0.600 |
| Q3 | 1.09 | 0.58, 2.05 | 0.800 | 0.95 | 0.48, 1.90 | 0.900 | 0.53 | 0.27, 1.04 | 0.063 |
| Q4 | 0.67 | 0.31, 1.44 | 0.300 | 0.74 | 0.31, 1.76 | 0.500 | 0.84 | 0.34, 2.12 | 0.700 |
| p for trend |  |  | 0.283 |  |  | 0.394 |  |  | 0.369 |
| Vitamin D |  |  |  |  |  |  |  |  |  |
| Q1 | Ref | — | — | Ref | — | — | Ref | — | — |
| Q2 | 1.29 | 0.84, 1.96 | 0.200 | 1.18 | 0.71, 1.95 | 0.500 | 1.24 | 0.73, 2.12 | 0.400 |
| Q3 | 2.03 | 1.23, 3.34 | 0.006 | 1.44 | 0.81, 2.58 | 0.200 | 1.47 | 0.82, 2.63 | 0.200 |
| Q4 | 1.82 | 0.70, 4.73 | 0.200 | 1.65 | 0.61, 4.44 | 0.300 | 1.04 | 0.35, 3.07 | >0.9 |
| p for trend |  |  | 0.131 |  |  | 0.262 |  |  | 0.859 |
| Vitamin K |  |  |  |  |  |  |  |  |  |
| Q1 | Ref | — | — | Ref | — | — | Ref | — | — |
| Q2 | 1.15 | 0.72, 1.84 | 0.500 | 0.75 | 0.46, 1.22 | 0.200 | 0.77 | 0.44, 1.35 | 0.400 |
| Q3 | 1.47 | 0.73, 2.96 | 0.300 | 1.41 | 0.66, 3.05 | 0.400 | 1.42 | 0.70, 2.88 | 0.300 |
| Q4 | 0.84 | 0.23, 3.10 | 0.800 | 0.75 | 0.19, 3.03 | 0.700 | 1.52 | 0.39, 5.96 | 0.500 |
| p for trend |  |  | 0.892 |  |  | 0.919 |  |  | 0.393 |

Model 1, no adjustment. Model 2, adjusted for age, race, BMI, education attainment, and ratio of family income to poverty. Model 3, adjusted for age, race, BMI, education attainment, ratio of family income to poverty, alcohol intake, smoking status, diabetes and hypertension. OR, odds ratio. CI, confidence interval.

Supplementary Table S4. Multivariate weighted logistic regression of the association between all vitamin intake quartiles and prostate cancer among US adults in the cross-sectional study.

| All vitamin intake type | Model 1 | | | Model 2 | | | Model 3 | | |
| --- | --- | --- | --- | --- | --- | --- | --- | --- | --- |
|  | OR*^1^* | 95% CI*^1^* | p | OR*^1^* | 95% CI*^1^* | p | OR*^1^* | 95% CI*^1^* | p |
| Vitamin B1 |  |  |  |  |  |  |  |  |  |
| Q1 | Ref | — | — | Ref | — | — | Ref | — | — |
| Q2 | 0.67 | 0.38, 1.16 | 0.150 | 0.86 | 0.47, 1.58 | 0.600 | 0.88 | 0.46, 1.68 | 0.700 |
| Q3 | 0.67 | 0.36, 1.24 | 0.200 | 0.97 | 0.48, 1.96 | >0.9 | 0.79 | 0.41, 1.55 | 0.500 |
| Q4 | 0.46 | 0.24, 0.86 | 0.016 | 0.68 | 0.35, 1.33 | 0.300 | 0.64 | 0.32, 1.30 | 0.200 |
| p for trend |  |  | 0.015 |  |  | 0.303 |  |  | 0.153 |
| Vitamin B2 |  |  |  |  |  |  |  |  |  |
| Q1 | Ref | — | — | Ref | — | — | Ref | — | — |
| Q2 | 0.50 | 0.29, 0.86 | 0.013 | 0.61 | 0.35, 1.08 | 0.089 | 0.72 | 0.38, 1.35 | 0.300 |
| Q3 | 0.60 | 0.33, 1.08 | 0.085 | 0.85 | 0.42, 1.70 | 0.600 | 0.89 | 0.43, 1.85 | 0.800 |
| Q4 | 0.40 | 0.23, 0.73 | 0.003 | 0.59 | 0.31, 1.14 | 0.120 | 0.66 | 0.34, 1.29 | 0.200 |
| p for trend |  |  | 0.005 |  |  | 0.223 |  |  | 0.325 |
| Vitamin B6 |  |  |  |  |  |  |  |  |  |
| Q1 | Ref | — | — | Ref | — | — | Ref | — | — |
| Q2 | 0.49 | 0.30, 0.83 | 0.008 | 0.55 | 0.32, 0.95 | 0.033 | 0.72 | 0.39, 1.34 | 0.300 |
| Q3 | 0.79 | 0.44, 1.43 | 0.400 | 0.98 | 0.51, 1.89 | >0.9 | 0.92 | 0.45, 1.85 | 0.800 |
| Q4 | 0.40 | 0.21, 0.75 | 0.005 | 0.53 | 0.26, 1.09 | 0.085 | 0.59 | 0.27, 1.28 | 0.200 |
| p for trend |  |  | 0.027 |  |  | 0.256 |  |  | 0.273 |
| Vitamin B9 |  |  |  |  |  |  |  |  |  |
| Q1 | Ref | — | — | Ref | — | — | Ref | — | — |
| Q2 | 1.12 | 0.70, 1.81 | 0.600 | 1.25 | 0.74, 2.12 | 0.400 | 0.89 | 0.49, 1.62 | 0.700 |
| Q3 | 0.83 | 0.44, 1.54 | 0.500 | 1.01 | 0.48, 2.14 | >0.9 | 0.99 | 0.43, 2.25 | >0.9 |
| Q4 | 0.65 | 0.32, 1.34 | 0.200 | 0.69 | 0.30, 1.59 | 0.400 | 0.57 | 0.23, 1.41 | 0.200 |
| p for trend |  |  | 0.160 |  |  | 0.329 |  |  | 0.271 |
| Vitamin B12 |  |  |  |  |  |  |  |  |  |
| Q1 | — | — |  | — | — |  | — | — |  |
| Q2 | 0.64 | 0.39, 1.04 | 0.071 | 0.71 | 0.42, 1.20 | 0.200 | 0.68 | 0.38, 1.23 | 0.200 |
| Q3 | 0.84 | 0.49, 1.45 | 0.500 | 0.72 | 0.40, 1.30 | 0.300 | 0.73 | 0.38, 1.40 | 0.300 |
| Q4 | 1.00 | 0.52, 1.89 | >0.9 | 0.84 | 0.42, 1.69 | 0.600 | 0.57 | 0.26, 1.26 | 0.200 |
| p for trend |  |  | 0.795 |  |  | 0.651 |  |  | 0.177 |
| Vitamin C |  |  |  |  |  |  |  |  |  |
| Q1 | Ref | — | — | Ref | — | — | Ref | — | — |
| Q2 | 1.02 | 0.59, 1.73 | >0.9 | 1.03 | 0.57, 1.85 | >0.9 | 0.98 | 0.52, 1.84 | >0.9 |
| Q3 | 1.27 | 0.78, 2.08 | 0.300 | 1.23 | 0.68, 2.22 | 0.500 | 1.05 | 0.60, 1.82 | 0.900 |
| Q4 | 0.87 | 0.50, 1.54 | 0.600 | 0.82 | 0.44, 1.53 | 0.500 | 0.67 | 0.35, 1.28 | 0.200 |
| p for trend |  |  | 0.841 |  |  | 0.673 |  |  | 0.255 |
| Vitamin D |  |  |  |  |  |  |  |  |  |
| Q1 | Ref | — | — | Ref | — | — | Ref | — | — |
| Q2 | 1.18 | 0.77, 1.80 | 0.400 | 0.99 | 0.61, 1.61 | >0.9 | 1.02 | 0.60, 1.71 | >0.9 |
| Q3 | 1.55 | 0.86, 2.81 | 0.140 | 0.95 | 0.47, 1.91 | 0.900 | 0.99 | 0.51, 1.89 | >0.9 |
| Q4 | 2.06 | 1.04, 4.10 | 0.039 | 1.88 | 0.97, 3.66 | 0.062 | 1.58 | 0.80, 3.12 | 0.200 |
| p for trend |  |  | 0.023 |  |  | 0.085 |  |  | 0.199 |
| Vitamin K |  |  |  |  |  |  |  |  |  |
| Q1 | Ref | — | — | Ref | — | — | Ref | — | — |
| Q2 | 1.20 | 0.68, 2.12 | 0.500 | 1.40 | 0.75, 2.61 | 0.300 | 1.20 | 0.60, 2.38 | 0.600 |
| Q3 | 1.50 | 0.85, 2.63 | 0.200 | 1.98 | 1.01, 3.90 | 0.048 | 1.38 | 0.67, 2.86 | 0.400 |
| Q4 | 0.77 | 0.42, 1.41 | 0.400 | 1.19 | 0.59, 2.40 | 0.600 | 1.13 | 0.52, 2.46 | 0.800 |
| p for trend |  |  | 0.548 |  |  | 0.436 |  |  | 0.668 |

Model 1, no adjustment. Model 2, adjusted for age, race, BMI, education attainment, and ratio of family income to poverty. Model 3, adjusted for age, race, BMI, education attainment, ratio of family income to poverty, alcohol intake, smoking status, diabetes and hypertension. OR, odds ratio. CI, confidence interval.

Supplementary Table S5. Model diagnostics and goodness-of-fit assessment for the logistic regression and RCS models.

| Type | Logistic model | | | | Restricted cubic spline model | | | | |
| --- | --- | --- | --- | --- | --- | --- | --- | --- | --- |
|  | Quartile analysis | | Continuous analysis | |  |  |  |  |  |
|  | Model 2 | Model 3 | Model 2 | Model 3 |  |  |  |  |  |
|  | VIF | VIF | VIF | VIF | VIF | Likelihood Ratio Test | C-index | Goodness-of-fit Test | |
|  |  |  |  |  |  | Chi-Square Value |  | Chi-Square Value | p |
| Dietary intake type |  |  |  |  |  |  |  |  |  |
| Vitamin A | 1.29 | 1.55 | 1.19 | 1.49 | 1.23 | 667.29 | 0.86 | 12.89 | 0.1157 |
| Vitamin B1 | 1.25 | 1.55 | 1.19 | 1.48 | 1.28 | 667.18 | 0.86 | 12.38 | 0.130 |
| Vitamin B2 | 1.24 | 2 | 1.21 | 1.49 | 1.32 | 667.26 | 0.86 | 12.27 | 0.140 |
| Vitamin B6 | 1.26 | 1.51 | 1.21 | 1.47 | 1.30 | 668.17 | 0.86 | 8.28 | 0.407 |
| Vitamin B9 | 1.22 | 1.49 | 1.2 | 1.44 | 1.30 | 667.72 | 0.86 | 9.02 | 0.341 |
| Vitamin B12 | 1.29 | 1.57 | 1.21 | 1.44 | 1.30 | 667.41 | 0.86 | 11.57 | 0.172 |
| Vitamin C | 1.29 | 1.51 | 1.28 | 1.49 | 1.31 | 667.34 | 0.86 | 11.58 | 0.171 |
| Vitamin D | 1.23 | 1.58 | 1.2 | 1.48 | 1.24 | 667.44 | 0.86 | 12.17 | 0.144 |
| Vitamin K | 1.25 | 1.49 | 1.17 | 1.43 | 1.24 | 667.24 | 0.86 | 12.15 | 0.145 |
| Supplement intake type |  |  |  |  |  |  |  |  |  |
| Vitamin B1 | 1.23 | 1.61 | 1.22 | 1.54 | 1.87 | 161.57 | 0.81 | 5.06 | 0.751 |
| Vitamin B2 | 1.27 | 1.51 | 1.2 | 1.5 | 2.17 | 162.83 | 0.81 | 7.54 | 0.479 |
| Vitamin B6 | 1.27 | 1.5 | 1.24 | 1.45 | 2.17 | 168.06 | 0.81 | 5.81 | 0.669 |
| Vitamin B9 | 1.24 | 1.48 | 1.18 | 1.42 | 2.15 | 169.79 | 0.81 | 1.72 | 0.989 |
| Vitamin B12 | 1.29 | 1.53 | 1.24 | 1.41 | 2.22 | 169.11 | 0.81 | 8.26 | 0.408 |
| Vitamin C | 1.28 | 1.49 | 1.26 | 1.46 | 2.16 | 183.81 | 0.81 | 7.62 | 0.471 |
| Vitamin D | 1.23 | 1.56 | 1.17 | 1.48 | 2.41 | 186.74 | 0.80 | 2.82 | 0.945 |
| Vitamin K | 1.19 | 1.45 | 1.15 | 1.41 | 2.38 | 139.02 | 0.81 | 3.05 | 0.931 |
| All intake type |  |  |  |  |  |  |  |  |  |
| Vitamin B1 | 1.22 | 1.63 | 1.23 | 1.53 | 2.03 | 161.53 | 0.81 | 6.77 | 0.562 |
| Vitamin B2 | 1.21 | 1.56 | 1.21 | 1.52 | 2.08 | 162.75 | 0.81 | 7.50 | 0.484 |
| Vitamin B6 | 1.27 | 1.48 | 1.22 | 1.48 | 2.15 | 168.07 | 0.81 | 8.15 | 0.419 |
| Vitamin B9 | 1.22 | 1.54 | 1.21 | 1.44 | 2.16 | 169.79 | 0.81 | 1.72 | 0.989 |
| Vitamin B12 | 1.3 | 1.52 | 1.2 | 1.45 | 2.16 | 168.99 | 0.81 | 8.26 | 0.409 |
| Vitamin C | 1.17 | 1.5 | 1.28 | 1.47 | 2.23 | 183.03 | 0.81 | 5.46 | 0.707 |
| Vitamin D | 1.18 | 1.55 | 1.19 | 1.49 | 2.40 | 186.46 | 0.80 | 2.42 | 0.965 |
| Vitamin K | 1.21 | 1.46 | 1.17 | 1.42 | 2.33 | 138.79 | 0.81 | 2.06 | 0.979 |

Model 2, adjusted for age, race, BMI, education attainment, and ratio of family income to poverty. Model 3, adjusted for age, race, BMI, education attainment, ratio of family income to poverty, alcohol intake, smoking status, diabetes and hypertension. VIF, variance inflation factor.
